# A Narrative Review of the Changing Landscape of Pediatric Practice in India: Public Health Triumphs and Professional Crises

**DOI:** 10.7759/cureus.99621

**Published:** 2025-12-19

**Authors:** Vikram Sakaleshpur Kumar

**Affiliations:** 1 Pediatrics and Child Health, Subbaiah Institute of Medical Sciences and Research Centre, Shivamogga, IND; 2 Pediatric Medicine, Sarji Mother and Child Care Hospital, Shivamogga, IND

**Keywords:** child health, healthcare delivery, india, lmic, medical education, pediatric workforce, physician compensation

## Abstract

Pediatric medicine developed as an independent medical discipline primarily responding to elevated infant mortality rates, prevalent infectious diseases, and childhood malnutrition. Effective immunization initiatives and enhanced nutritional standards have substantially decreased these conventional disease burdens, especially across low- and middle-income countries (LMICs), such as India. This epidemiological shift has generated fundamental challenges for pediatric medicine as a specialized field, evidenced by waning medical student engagement, personnel shortages, and consistently modest remuneration relative to other medical specialties. This review investigates the evolving pediatric practice environment in India relative to other LMICs and developed nations (the United States (US), the United Kingdom (UK), Europe, and Australia), examines workforce dynamics and compensation patterns through evidence-based analysis, and suggests strategic pathways for the discipline's advancement to maintain relevance and viability. The analysis utilizes a narrative review methodology incorporating a comprehensive evaluation of peer-reviewed literature, governmental publications, and workforce analyses published between 2015 and 2025. India's infant mortality rate decreased from 88 in 1990 to 27 per 1,000 live births in 2021, representing a 69% reduction, while the Under-5 Mortality Rate declined from 126 to 32 per 1,000 live births - a 75% decrease. These achievements have fundamentally transformed pediatric practice patterns but created workforce challenges, including declining medical student interest, with only 8.3% expressing pediatric preferences compared to 24.7% for internal medicine. Compensation analysis reveals pediatricians earn 35-40% less than other specialists in comparable practice settings. International comparisons demonstrate similar patterns across high-income countries, with American pediatricians ranking among the lowest-paid specialists and the UK reporting 25% consultant vacancy rates. Strategic recommendations include educational reform emphasizing non-communicable diseases and mental health, practice model innovation incorporating integrated care approaches, policy advocacy for appropriate reimbursement structures, and career diversification opportunities. Success requires coordinated efforts across medical education, practice innovation, policy development, and professional advocacy to demonstrate pediatric medicine's continued value in addressing contemporary child health challenges while maintaining specialty sustainability.

## Introduction and background

The emergence of pediatric medicine as an autonomous medical subspecialty during the early to mid-20th century stemmed from two paramount public health concerns: the catastrophic impact of infectious diseases and pervasive childhood undernutrition. These conditions constituted the predominant sources of pediatric morbidity and mortality worldwide, requiring specialized knowledge in child health management [1,2].

Within the Indian context, the historical circumstances were especially dire. Upon achieving independence in 1947, India's infant mortality rate (IMR) surpassed 150 deaths per 1,000 live births, with infectious conditions, including measles, diphtheria, pertussis, and diarrheal diseases, representing the primary causes of childhood mortality [3]. Protein-energy malnutrition affected substantial numbers of children, exacerbating infectious disease vulnerability and clinical outcomes [4].

The preceding three to four decades have demonstrated extraordinary advancement. India's IMR decreased from 88 in 1990 to 27 in 2021, constituting a 69% reduction [5,6]. The Under-5 Mortality Rate (U5MR) declined from 126 to 32 per 1,000 live births during this timeframe - representing a 75% decrease. Correspondingly, the neonatal mortality rate (NMR) decreased from 56 to 19 per 1,000 live births between 1990 and 2021 [5,7]. These achievements, primarily attributable to enhanced immunization coverage through the Universal Immunization Programme (UIP) and strengthened nutritional interventions, have fundamentally transformed pediatric practice patterns [8,9].

Nevertheless, this epidemiological transformation has produced an unforeseen consequence: the foundational justification for pediatric medicine as a specialized discipline has been considerably weakened. In the Indian context, pediatric medicine encompasses newborn care, well-baby care, acute and chronic disease management, developmental and behavioural pediatrics, adolescent health, and community-oriented interventions, such as national immunization programmes, school health services, and child-survival initiatives. The discipline is guided by national policies (NMC regulations, National Health Mission, Universal Immunization Programme) and professional standards laid down by the Indian Academy of Pediatrics (IAP).

The field currently confronts significant challenges, including diminishing medical student attraction, workforce deficits, inadequate compensation compared to other specialties, and uncertainty regarding future relevance. This phenomenon extends beyond India, manifesting across numerous countries in both LMIC and high-income settings [10,11].

The purpose of this review is to examine the changing landscape of pediatric practice across different economic contexts, analyze current workforce and compensation challenges, and propose strategic directions for the specialty's evolution to ensure continued relevance and sustainability in the contemporary healthcare environment.

## Review

Methodology

A narrative review methodology was used to synthesize evidence on epidemiology, pediatric workforce dynamics, compensation patterns, and medical education trends across India, other LMICs, and high-income countries. Searches were conducted in PubMed, Scopus, Google Scholar, and government or professional-body repositories (NFHS, UNIGME, WHO Global Health Workforce Statistics, NHS Workforce Data, AAP, Medscape, AMA) for publications between 2015 and 2025. Search terms included “pediatric workforce”, “pediatrician compensation”, “child mortality India”, “pediatric residency trends”, “LMIC pediatric workforce”, and “medical specialty preferences”. English-language peer-reviewed studies, national reports, and workforce analyses were included. Editorials without data, commentary pieces, and non-health-system opinion articles were excluded. Sources were evaluated for relevance to epidemiology, workforce density, vacancy rates, compensation, or training patterns. Findings were integrated narratively, supported by tables and figures summarizing key trends.

Current landscape of pediatric practice in India

Data Sources and Population Context

To provide population-level context, national birth and mortality datasets were reviewed to estimate the scope of pediatric health needs during the study period. India recorded approximately 23-25 million live births annually between 2015 and 2021, representing one of the largest newborn cohorts globally. From the literature and national databases screened, 68 records (peer-reviewed studies, government reports, and workforce analyses) were identified as relevant and included in the synthesis. These sources provided epidemiological indicators, workforce statistics, and pediatric service trends across India and comparator countries. While narrative reviews do not employ formal PRISMA flow diagrams, the number of records incorporated and the underlying national birth volumes help contextualize the scale of the pediatric population analyzed.

Epidemiological Transformation

India's epidemiological transition represents one of the most remarkable public health achievements of the late 20th and early 21st centuries. The dramatic reduction in childhood mortality has been accompanied by substantial changes in disease patterns affecting pediatric practice.

**Figure 1:**
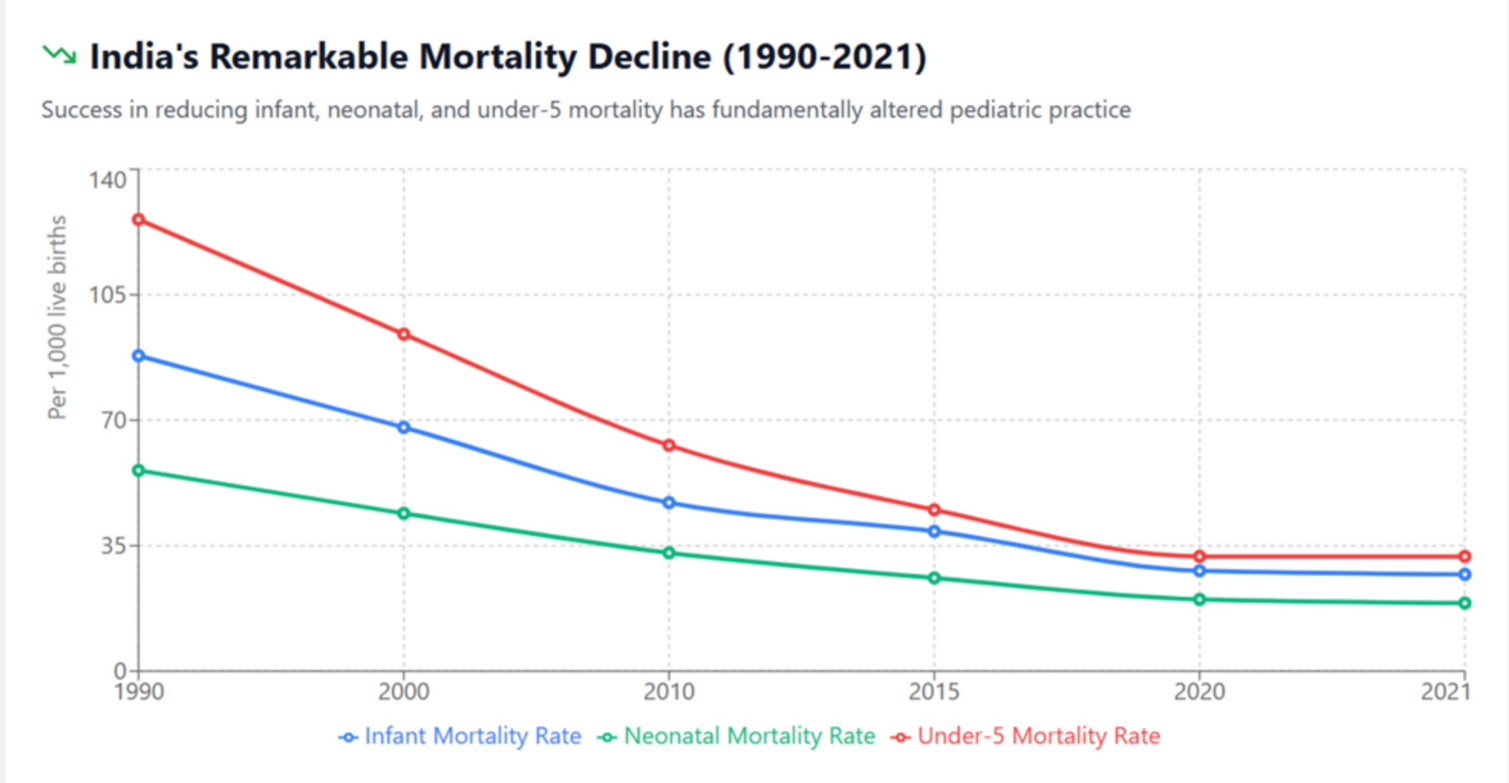
India's Child Mortality Decline (1990-2021) The figure demonstrates India's remarkable success in reducing childhood mortality over three decades. The under-5 mortality rate decreased by 75% (from 126 to 32 per 1,000 live births), infant mortality rate by 69% (from 88 to 27), and neonatal mortality rate by 66% (from 56 to 19). These dramatic improvements have fundamentally altered the traditional disease burden that historically justified pediatrics as a distinct specialty.

See Table 1 for comprehensive India mortality trends. Figure 1 visually demonstrates the remarkable 66-75% reductions across all mortality indicators.

**Table 1:** India's Epidemiological Transformation: Key Child Health Indicators (1990-2021) India has achieved remarkable reductions in infant, neonatal, and under-5 mortality rates over three decades, with immunization coverage more than doubling. However, anemia increased from NFHS-4 to NFHS-5, indicating persistent nutritional challenges despite overall mortality improvements. Source: NFHS-3, NFHS-4, NFHS-5, and UNIGME 2024 Reports [5,7]

Indicator	1990	2000	2010	2015	2020	2021	% Decline (1990-2021)
Infant Mortality Rate (per 1,000 live births)	88	68	47	39	28	27	69%
Neonatal Mortality Rate (per 1,000 live births)	56	44	33	26	20	19	66%
Under-5 Mortality Rate (per 1,000 live births)	126	94	63	45	32	32	75%
Full Immunization Coverage (%)	35.4	42	61	62	75	76.3	116%
Stunting Prevalence (%)	51.6	48	41.6	38.4	--	35.5	31%
Childhood Anemia (%)	74	70	69.5	58.6	--	67.1	9%

The implementation of comprehensive vaccination programs has virtually eliminated several childhood diseases that previously required specialized pediatric expertise. Vaccine-preventable diseases such as poliomyelitis, measles, diphtheria, and pertussis have experienced dramatic incidence reductions [12,13]. The introduction of newer vaccines, including Haemophilus influenzae type b (Hib), pneumococcal conjugate vaccine (PCV), and rotavirus vaccine, has further reduced hospitalization rates for previously common pediatric conditions [14,15].

Concurrent improvements in childhood nutritional status have been documented across India. The National Family Health Survey (NFHS-5) data indicate significant reductions in stunting, wasting, and underweight prevalence among children under five years compared to previous surveys [16]. These improvements have reduced the traditional burden of malnutrition-related complications that historically constituted a substantial portion of pediatric practice.

Workforce Challenges

Recent studies examining medical student specialty preferences in India demonstrate concerning trends for pediatrics. A multi-institutional survey conducted across medical colleges revealed that pediatrics ranked among the least preferred specialties, with only 8.3% of students expressing interest compared to 24.7% for internal medicine and 18.2% for surgery [17]. Factors contributing to this decline include perceived lower earning potential, emotional stress associated with caring for sick children, and limited career advancement opportunities. The premier institutions report increasing difficulty in filling pediatric residency positions. Data from the National Eligibility cum Entrance Test (NEET-PG) indicate that pediatrics consistently has lower cut-off scores compared to other clinical specialties, suggesting reduced competition and interest [18]. Pediatric workforce distribution in India demonstrates significant urban-rural disparities. Urban centers, particularly metropolitan areas, have relatively adequate pediatric coverage, while rural and semi-urban regions face severe shortages. The Indian Academy of Pediatrics estimates that over 60% of India's pediatricians practice in urban areas, serving less than 35% of the child population [19].

Compensation Patterns

Compensation analysis reveals that pediatric practitioners earn significantly less than colleagues in other specialties. A comprehensive salary survey conducted by a few associations through digital platforms indicated that pediatricians earn approximately 35-40% less than orthopedic surgeons, cardiologists, or gastroenterologists in comparable practice settings [20]. This disparity exists across both private practice and institutional employment contexts.

The economics of pediatric practice present unique challenges. Consultation fees for pediatric services are generally lower than those of adult medicine, partly due to parental expectations and social perceptions of child healthcare as a basic service rather than specialized expertise. Additionally, the emotional burden of caring for sick children and dealing with anxious parents contributes to professional stress without commensurate financial compensation [21].

International comparisons

High-Income Countries

The American pediatric workforce faces similar challenges despite different healthcare system structures. The American Academy of Pediatrics (AAP) reports persistent concerns about pediatric workforce adequacy, particularly in subspecialty areas. Compensation disparities remain significant, with pediatricians earning substantially less than other specialists [22]. The 2023 Medscape Physician Compensation Report indicated that pediatricians ranked among the lowest-paid specialists, with average annual incomes approximately 40% lower than those of orthopedic surgeons [22-24].

**Figure 2:**
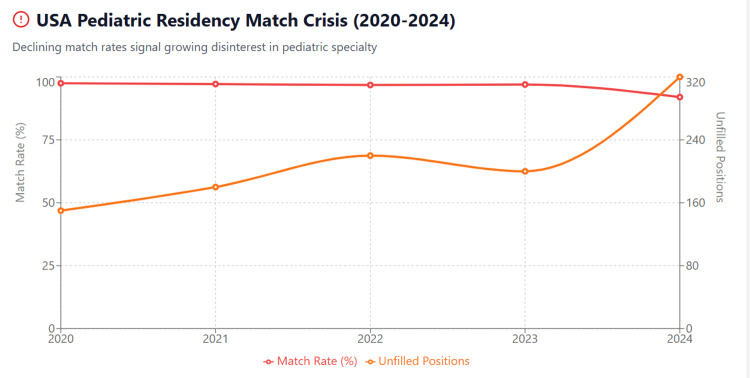
USA Pediatric Residency Crisis: Match Rates and Unfilled Positions (2020-2024) The figure illustrates the accelerating pediatric workforce crisis in the USA. The 2024 match rate plummeted to 92% from 97% in 2023—the largest single-year decline in a decade. Unfilled positions nearly tripled from 90 to 241, with nearly one-third of pediatric residency programs unable to fill all positions. Applications dropped 6% year-over-year, signaling declining medical student interest.

Table 2 details the year-by-year decline in pediatric residency match rates. Figure 2 illustrates the dramatic 2024 crisis with 241 unfilled positions representing a 168% increase from 2023 [23].

**Table 2:** Pediatric Residency Match Trends in the USA (2020-2024) The 2024 pediatric residency match revealed a crisis, with match rates dropping to 92% from 97% in 2023. Nearly one-third of programs failed to fill all positions, and applications dropped 6% year-over-year—the largest single-year decline in a decade. Source: National Resident Matching Program (NRMP) Main Match Data 2020-2024 [23]

Year	Total Positions	Positions Filled	Match Rate (%)	Applications	Applications per Position	Unfilled Positions
2020	2,835	2,762	97.50%	4,250	1.5	73
2021	2,901	2,820	97.20%	4,195	1.45	81
2022	2,945	2,851	96.80%	4,120	1.4	94
2023	2,987	2,897	97.00%	4,085	1.37	90
2024	3,012	2,771	92.00%	3,840	1.27	241

**Figure 3:**
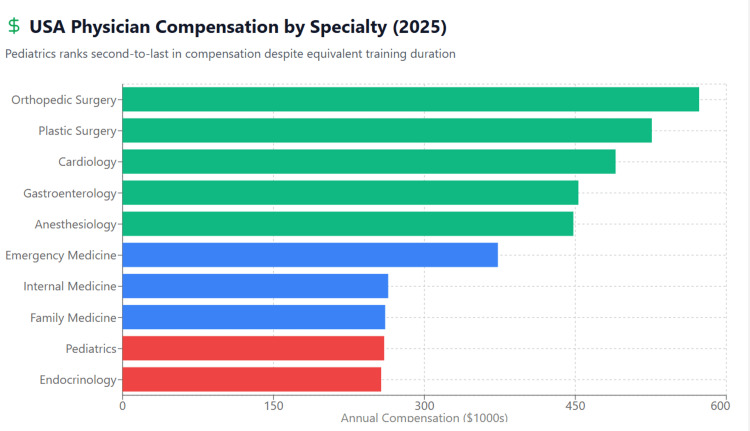
Physician Compensation Hierarchy: Pediatrics at the Bottom (USA, 2025) The figure demonstrates pediatrics' position at the bottom of the physician compensation hierarchy, ranking 28th out of 29 specialties. At $260,000 annually, pediatricians earn 28% below the overall physician average and 55% less than orthopedic surgeons, despite equivalent training duration. This substantial compensation disparity represents a critical driver of declining student interest in pediatrics.

Table 3 ranks all medical specialties by compensation, demonstrating pediatrics' position near the bottom. Figure 3 provides a visual comparison showing that pediatrics earns approximately $313,000 less than the top-earning specialties.

**Table 3:** Physician Compensation Comparison by Specialty in the USA (2025) Pediatrics ranks 28th out of 29 specialties in compensation, earning 28% below the physician average and $313,000 less than orthopedic surgery. Despite equivalent training duration, pediatricians earn substantially less than most other specialties. Source: Medscape Physician Compensation Report 2025 [23-25]

Specialty	Average Annual Compensation	% vs Physician Average	Rank (of 29)
Orthopedic Surgery	$573,000	58%	1
Plastic Surgery	$526,000	45%	2
Cardiology	$490,000	35%	3
Gastroenterology	$453,000	25%	4
Anesthesiology	$448,000	23%	5
Radiology	$437,000	20%	6
Emergency Medicine	$373,000	3%	12
Internal Medicine	$264,000	-27%	26
Family Medicine	$261,000	-28%	27
Pediatrics	$260,000	-28%	28
Endocrinology	$257,000	-29%	29
Overall Physician Average	$363,000	--	--

**Figure 4:**
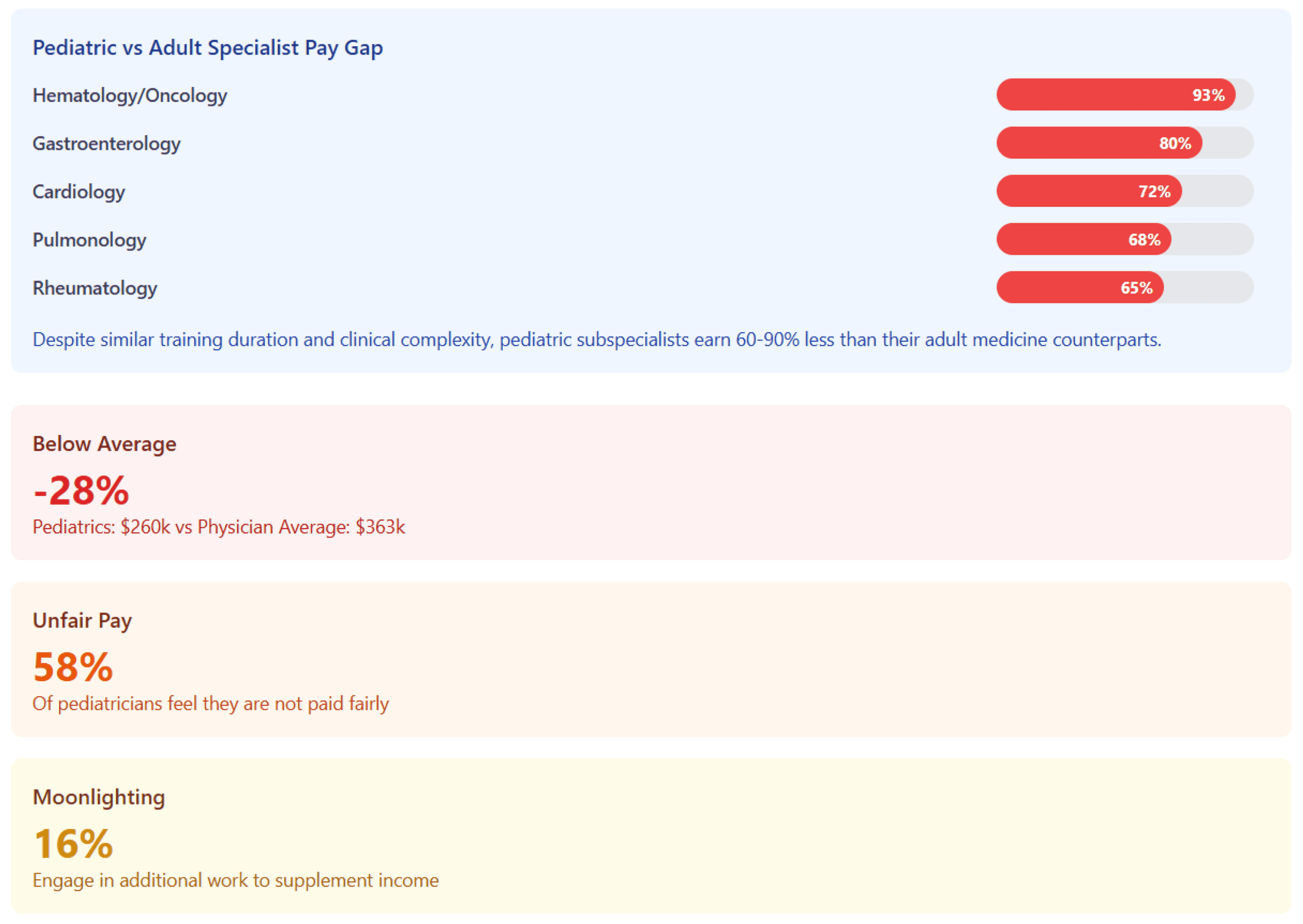
Pediatric vs Adult Medicine Subspecialty Pay Gaps The figure reveals systematic compensation disparities between pediatric and adult medicine subspecialties. Despite comparable training and clinical complexity, pediatric subspecialists earn 44-93% less than adultmedicine counterparts. These gaps translate to lifetime earning differences of $1.6-4.1 million, creating powerful financial disincentives for pediatric specialists.

Table 4 and Figure 4 detail the systematic pay gaps between pediatric and adult medicine subspecialties, ranging from 44% to 93%.

**Table 4:** Pediatric vs Adult Medicine Subspecialty Pay Gaps (2025) Despite similar training duration and complexity of care, pediatric subspecialists earn 44-93% less than their adult medicine counterparts. Lifetime earning differences range from $1.6 million to over $4 million, contributing significantly to declining interest in pediatric subspecialties. Source: Catenaccio et al. [26,27]; Doximity 2025 Physician Compensation Report [28]

Subspecialty Field	Adult Medicine Compensation	Pediatric Compensation	Pay Gap (%)	Lifetime Earnings Difference
Hematology/Oncology	$425,000	$220,000	93%	$4,100,000
Gastroenterology	$453,000	$252,000	80%	$4,020,000
Cardiology	$490,000	$285,000	72%	$4,100,000
Pulmonology	$408,000	$243,000	68%	$3,300,0002
Rheumatology	$298,000	$181,000	65%	$2,340,000
Endocrinology	$257,000	$178,000	44%	$1,580,000

Across other high-income countries, pediatric workforce challenges reveal both shared and region-specific patterns. In the United Kingdom, the National Health Service (NHS) continues to face acute pediatric workforce shortages. The Royal College of Paediatrics and Child Health reports a 25% vacancy rate among pediatric consultants and a 15% vacancy rate among pediatric trainees [25]. These deficits have been intensified by post-Brexit workforce departures, which have particularly affected subspecialty services. Although pediatricians receive base salaries comparable to other NHS consultants, opportunities for private practice are limited, restricting overall earning potential. The demanding nature of pediatric work, marked by frequent out-of-hours responsibilities and considerable emotional stress, further contributes to professional dissatisfaction [29].

Similar variations are evident across the European Union, where workforce strength and training infrastructure differ substantially between regions. Countries such as Germany and France maintain relatively robust pediatric training programs, whereas Southern and Eastern European nations struggle with persistent shortages. A survey by the European Academy of Paediatrics found that over 60% of member countries reported inadequate pediatric staffing [30]. Compensation disparities across Europe mirror these differences: pediatricians typically earn less than surgical or high-income internal medicine subspecialists. Nonetheless, the presence of stronger social safety nets and alternative practice models in several European systems provides partial mitigation of income gaps and burnout pressures [31].

In Australia, pediatric practice demonstrates a familiar urban-rural imbalance. Metropolitan regions are generally well served, while rural and remote areas remain significantly underserved. The Royal Australasian College of Physicians notes that interest in pediatric training has remained steady, though modest, over recent years [32]. Australian pediatricians earn competitively within the broader medical profession; however, remuneration continues to lag behind procedural specialties. The Medicare Benefits Schedule ensures standardized compensation but inherently limits income growth compared to more procedure-driven disciplines [33,34].

Other Low- and Middle-Income Countries

**Figure 5:**
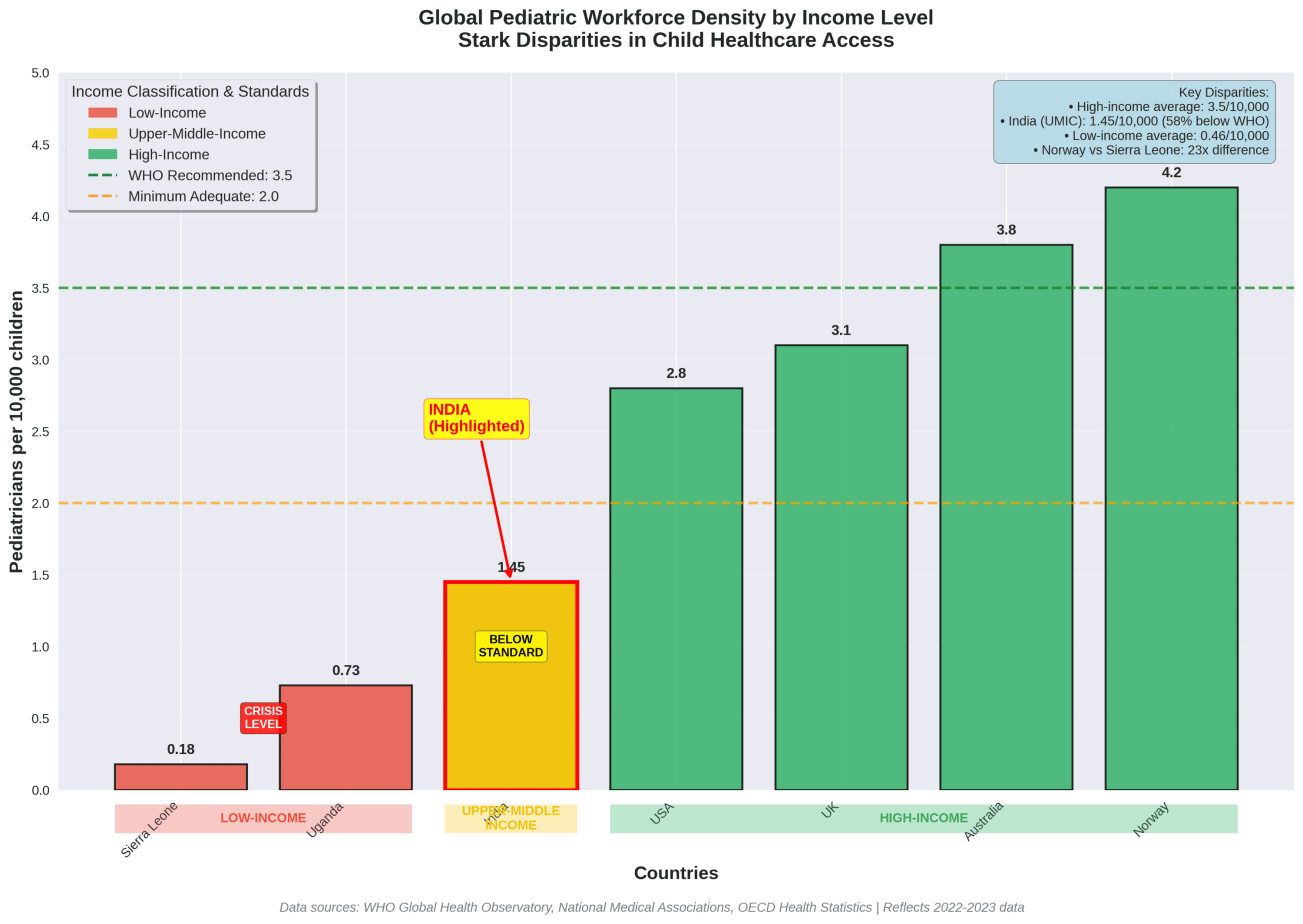
Global Pediatric Workforce Density The figure compares pediatric workforce density across countries at different income levels. High-income countries maintain 2.8-4.2 pediatricians per 10,000 children, though most still face workforce shortages. India, as a representative LMIC, has 1.45 pediatricians per 10,000 children—below adequate levels but substantially better than low-income countries with ratios as low as 0.18 per 10,000.

Across low- and middle-income countries, pediatric workforce shortages present some of the most critical global health challenges. Nowhere is this crisis more pronounced than in sub-Saharan Africa, where the World Health Organization estimates fewer than 0.5 pediatricians per 100,000 children - far below the recommended ratio of 1.5-2.0 per 100,000. Nations such as Nigeria, Kenya, and Ghana continue to lose trained pediatricians to high-income countries, deepening already fragile local healthcare systems [34]. Table 5 and Figure 5 illustrate the stark contrasts in workforce density across income levels, positioning India in an intermediate zone between well-resourced nations and low-income countries with near-critical shortages.

**Table 5:** Global Pediatric Workforce Density Comparison Pediatric workforce density varies dramatically across income levels. High-income countries maintain 2.8-4.2 pediatricians per 10,000 children but still face vacancy rates of 8-19%. LMICs have critical shortages, with some countries having fewer than 1 pediatrician per 10,000 children or zero pediatric surgeons. Source: WHO Global Health Workforce Statistics 2023 [33]; National Workforce Reports 2024 [34]

Country/Region	Pediatricians per 10,000 Children	Overall Physician Density (per 1,000 population)	Pediatric Vacancy Rate (%)	Classification
Norway	4.2	5	8%	High-income
Australia	3.8	3.8	12%	High-income
United Kingdom	3.1	3	19.2% (CAMHS)	High-income
United States	2.8	2.6	8% (overall)	High-income
India	1.45	0.9	Unknown	LMIC
Uganda	0.73	0.3	45%+	LMIC
Sierra Leone	0.18	0.2	60%+	LMIC

Despite shouldering higher disease burdens than India, many sub-Saharan African nations face similar structural barriers to developing a stable pediatric workforce. Limited compensation, inadequate facilities, and strenuous working conditions deter medical graduates from choosing pediatrics as a career [35]. Southeast Asian countries present a more varied picture. Thailand, for instance, has mirrored India’s success in improving child health outcomes, but these gains have brought their own workforce pressures. Malaysia has sustained a relatively balanced pediatric workforce through government-backed employment guarantees, while Indonesia continues to grapple with acute shortages in rural regions [36,37].

In Latin America, workforce dynamics show further diversity. Brazil’s unified health system offers job security for pediatricians but struggles with equitable distribution, particularly outside major cities. Mexico’s dual public-private healthcare model has created sharp compensation gaps that influence specialty choice, while Argentina’s pediatric sector faces fluctuating employment stability depending on regional economic conditions [26,38].

Emerging challenges and opportunities

Changing Disease Patterns

The epidemiological transition has shifted pediatric disease patterns toward non-communicable conditions. Childhood obesity, diabetes mellitus type 2, hypertension, and mental health disorders are increasingly prevalent [27,39,40]. These conditions require different skill sets and practice approaches compared to traditional infectious diseases and malnutrition.

**Figure 6:**
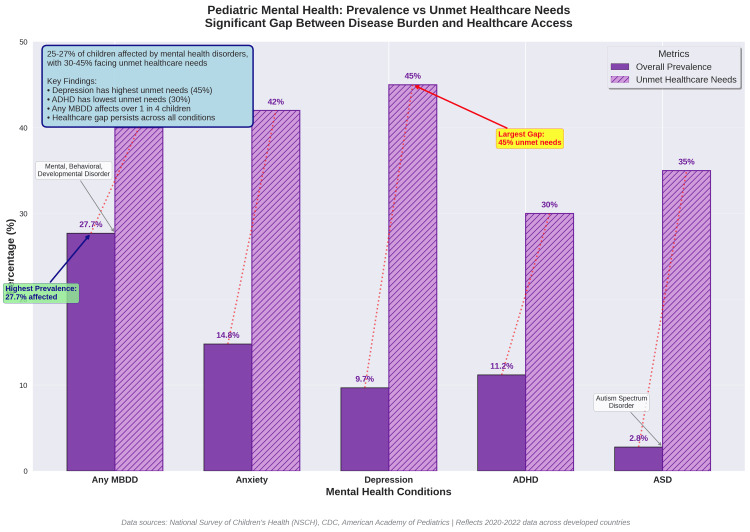
Childhood Mental Health Crisis: Prevalence and Unmet Needs The figure demonstrates the pediatric mental health crisis. Mental, behavioral, and developmental disorders affect 25-27% of children, with 30-45% experiencing unmet healthcare needs. Depression and anxiety show the highest gaps between prevalence and access to care (42-45% unmet needs), highlighting the urgent need for expanded pediatric mental health services and training.

Emerging patterns in child health reveal a striking shift in the global disease landscape. Table 6 summarizes the evolving burden of pediatric conditions, while Figure 6 highlights that 25-27% of children are affected by mental health disorders, with an estimated 30-45% of these cases remaining untreated. This widening gap between prevalence and access underscores one of the most urgent challenges in modern pediatrics.

**Table 6:** Emerging Disease Burden: Mental Health and Chronic Diseases in Children Traditional infectious diseases have declined, but mental health disorders now affect over 25% of children, with 40-45% facing unmet healthcare needs. Childhood obesity and associated chronic diseases represent emerging epidemics requiring specialized pediatric expertise distinct from historical disease burdens.

Condition Category	Prevalence (%)	Year-over-Year Trend	Healthcare Access Gap	Primary Source
Mental, Behavioral, Developmental Disorders	25.3-27.7%	↑ Increasing	40% unmet needs	Leeb et al. 2024
Depression	4.4-9.7%	↑ Increasing	45% untreated	Xiang et al. 2024
Anxiety Disorders	9.2-14.8%	↑ Increasing	42% untreated	Racine et al. 2021
ADHD	9.8-11.2%	↑ Increasing	30% undiagnosed	Leeb et al. 2024
Autism Spectrum Disorder	2.3-2.8%	↑ Increasing	35% delayed diagnosis	CDC 2023
Childhood Obesity	19.7% (USA), 2-7% (India)	↑ Increasing	Limited intervention	Di Cesare et al. 2019
Type 2 Diabetes	0.67-0.95 per 1,000	↑ Increasing	25% undiagnosed	Kumar & Kelly 2017
Asthma (obesity-related)	8.4-13.2%	↑ Increasing	Variable control	Lang et al. 2018

**Figure 7:**
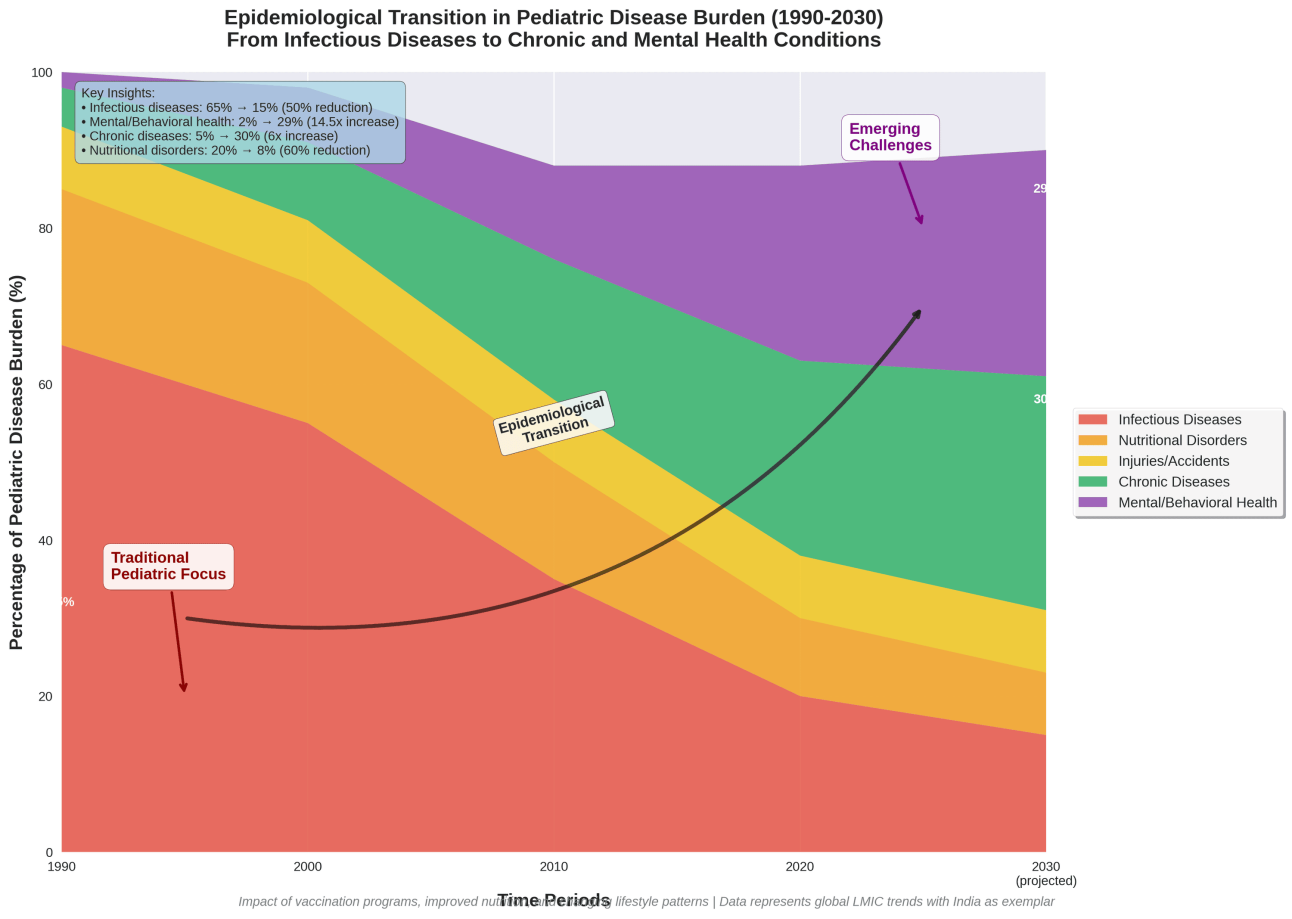
The Transformation of Pediatric Disease Burden The figure illustrates the dramatic epidemiological transition in pediatric disease burden from 1990-2030 (projected). Traditional infectious diseases and nutritional disorders, which historically justified pediatrics as a distinct specialty, have declined from 85% to 23% of the disease burden. Conversely, mental/behavioral health disorders and chronic diseases have increased from 7% to 59%, requiring fundamentally different clinical expertise and practice models.

Neurodevelopmental and behavioral disorders have become increasingly prominent. Autism spectrum disorder, attention deficit hyperactivity disorder (ADHD), learning disabilities, and other developmental conditions are being recognized and diagnosed with growing frequency [39]. Managing these disorders requires long-term, multidisciplinary approaches that differ fundamentally from traditional models of acute infectious disease care [40]. Figure 7 depicts the broader epidemiological transition, from infectious diseases accounting for 65% of the pediatric disease burden in 1990 to an estimated 59% dominated by mental health and chronic conditions by 2030; capturing the paradox at the heart of contemporary pediatric practice.

Parallel to these neurodevelopmental trends, global childhood obesity has emerged as a major public health concern. Figure 8 illustrates this trajectory, showing that approximately 340 million children are currently affected, with prevalence projected to rise across all regions by 2030. The metabolic, cardiovascular, and psychosocial consequences of this trend foreshadow an expanded role for pediatricians in preventive and lifestyle-focused medicine.

The mental health dimension of child health has also intensified, particularly in the aftermath of the COVID-19 pandemic. Pediatricians now confront rising rates of anxiety, depression, and behavioral disorders that require close collaboration with psychiatrists, psychologists, educators, and community health workers [41,42]. Together, these developments signal an irreversible transformation in the scope of pediatric care-one that demands new competencies, broader interprofessional partnerships, and sustained systemic attention.

Technological Integration

The COVID-19 pandemic served as a catalyst for the rapid integration of telemedicine into pediatric care. What began as a necessity for infection control has evolved into a lasting feature of clinical practice. Remote consultations, digital monitoring, and virtual follow-up visits now form essential components of pediatric service delivery, particularly in rural and underserved areas where access to specialists remains limited [43-45].

Beyond telemedicine, the digital transformation of pediatric healthcare continues to gather pace [45]. Electronic health records, mobile health applications, and wearable monitoring devices are reshaping how pediatricians document, communicate, and coordinate care. These technologies enable more continuous engagement with patients and families while improving data-driven population health management [46].

At the same time, rapid advances in genomics, pharmacogenomics, and personalized medicine are redefining the frontiers of pediatric specialization. Precision-based approaches offer the potential to tailor prevention and therapy to each child’s biological profile. Realizing this potential, however, will require a generation of pediatricians trained in data interpretation, molecular diagnostics, and interdisciplinary collaboration; skills that collectively strengthen the evolving value proposition of pediatric practice [47,48].

Strategic recommendations for specialty evolution

Educational Reform

Reforming pediatric education is central to preparing the next generation of clinicians for a rapidly changing health landscape. Training programs must evolve to address contemporary challenges that extend far beyond infectious disease management. Curricula should emphasize non-communicable diseases, mental health, developmental disorders, and population health approaches. Equally important is the integration of technology, quality improvement principles, and healthcare delivery science as standard components of pediatric education [49,50].

The implementation of competency-based medical education (CBME) frameworks offers a path toward greater adaptability and relevance. By focusing on demonstrable skills rather than time-based progression, CBME enables trainees to develop competencies aligned with the realities of modern practice. This flexibility also allows pediatric residents to pursue diverse career interests across clinical care, research, health systems, and advocacy within a coherent structure [51,52].

Modern pediatric training must also reflect the collaborative nature of child health. Interprofessional education that brings together nurses, social workers, psychologists, and mental health professionals is essential for building cohesive care teams. Such integration strengthens communication, mutual respect, and shared accountability, which are fundamental to effective pediatric practice in increasingly complex healthcare settings [53,54].

Practice Model Innovation

Innovating pediatric practice models is essential to sustain the specialty and improve child health outcomes. The development of integrated care systems that combine traditional pediatric services with mental health, nutrition, developmental, and family support components can create more comprehensive and sustainable frameworks of care. Such models respond to the holistic needs of children and families while offering pediatricians more meaningful and balanced professional roles [55,56].

Pediatric practice must also evolve beyond individual patient encounters to engage with broader public health goals. Involvement in population health programs, community health promotion, and preventive care initiatives can reinforce the specialty’s relevance and strengthen its social value [57,58].

Further growth lies in the strategic development of new pediatric subspecialties. Fields such as obesity medicine, behavioral health, adolescent medicine, and global health offer opportunities for diversification, professional advancement, and improved financial stability. Expanding training and research within these areas will help align pediatric practice with emerging global health priorities [59,60].

Policy and Advocacy

Sustaining the pediatric workforce requires advocacy that reinforces the essential role of child health expertise within the broader healthcare system. Pediatricians should actively support policies that recognize this value through appropriate reimbursement structures, quality indicators that reflect the complexity of pediatric care, and workforce planning that addresses regional disparities [61,62].

Professional organizations also have a crucial responsibility in strengthening public recognition of pediatrics as a specialized and indispensable discipline. Strategic communication through education campaigns and media engagement can help sustain societal appreciation for pediatric expertise, particularly as health priorities shift over time [63,64].

Ongoing investment in pediatric research remains fundamental to maintaining the specialty’s relevance and impact. Research efforts should target current health challenges, innovative models of care delivery, and robust outcomes evaluation to demonstrate measurable benefits for children and families [65,66].

Compensation and career sustainability

Sustainable pediatric practice depends on compensation structures that accurately reflect the scope and value of child health services. Healthcare systems should explore alternative payment models such as capitation, quality-based reimbursement, and population health incentives to create fairer and more stable financial frameworks for pediatric care [67,68].

Pediatricians should also be encouraged to cultivate diverse professional portfolios that integrate clinical service, research, teaching, administration, and public health engagement. This diversification not only enhances career satisfaction but also strengthens financial resilience across different healthcare environments [69,70].

As documented in Table 7 and previously in Tables 3-4, pediatrician compensation varies widely among countries but remains consistently lower than that of most other medical specialties. Satisfaction with remuneration is correspondingly low, with fewer than 45 percent of pediatricians in high-income settings such as the United States and the United Kingdom reporting that they feel fairly compensated. This persistent disparity underscores the need for systemic reforms that align payment with both the complexity and societal value of pediatric care [71].

**Table 7:** Pediatrician Compensation and Satisfaction Metrics by Country Pediatrician compensation varies significantly across countries but consistently ranks at the bottom of specialty hierarchies. Satisfaction with compensation is low across high-income countries, with less than 45% of US and UK pediatricians feeling fairly compensated. Source: Medscape 2025 [36], NHS Workforce Data 2024 [32], and Australian Medical Association 2024 [34]

Country	Entry-Level Annual Salary (USD)	Mid-Career Salary (USD)	Senior-Level Salary (USD)	Fair Pay Perception (%)	Job Satisfaction (%)
United States	$180,000-$220,000	$240,000-$280,000	$280,000-$350,000	42%	58%
United Kingdom	$55,000-$75,000	$90,000-$120,000	$130,000-$180,000	38%	52%
Australia	$85,000-$110,000	$140,000-$180,000	$200,000-$280,000	56%	68%
India	$6,000-$10,000	$10,000-$15,000	$15,000-$25,000	Unknown	Unknown

Global Pediatric Workforce Comparison

To contextualize India’s workforce challenges within broader international patterns, a comparative summary of pediatric workforce indicators across economic settings is presented in Table 8. This table integrates pediatrician density, vacancy rates, compensation positioning, and structural workforce issues across high-income countries, other LMICs, and low-income regions. The synthesis highlights that although India occupies a mid-range position globally, the specialty faces common pressures seen worldwide: declining trainee interest, uneven distribution, and persistent under-compensation, underscoring the need for coordinated reforms across education, policy, and service delivery.

**Table 8:** Comparative Pediatric Workforce Indicators Across Economic Settings (India, LMICs, High-Income Countries) Data synthesized from WHO 2023 [22,40,45], NHS Workforce 2024 [32], NRMP 2024 [23], AMA/Medscape 2025 [36], national workforce reports [34-38], and the present review’s findings

Country/Region	Pediatricians per 10,000 Children	Vacancy Rate/Unfilled Posts	Compensation Ranking	Workforce Challenges
India (LMIC)	1.45	Not formally reported; significant rural deficits	Low relative to specialties in India	Urban–rural maldistribution; declining student interest; low remuneration
United States (HIC)	2.8	241 unfilled residency posts in 2024; match rate 92%	28th of 29 specialties	Large subspecialty shortages; lowest-paid specialists; declining applicants
United Kingdom (HIC)	3.1	25% consultant vacancies; 15% trainee vacancies	Salary comparable to NHS specialists but limited private income	Shortages worsened post-Brexit; high on-call burden
Australia (HIC)	3.8	Shortages mainly in rural areas	Competitive but below procedural specialties	Geographic disparities; capped Medicare remuneration
Europe (Mixed HIC)	2.5–4.0 (varies by region)	>60% of countries report inadequate staffing	Moderate	Southern/Eastern Europe face chronic shortages
Sub-Saharan Africa (Low-income)	0.18–0.73	Vacancy rates >45–60%	Very low	Severe shortages, migration to HICs, limited training capacity
Latin America (Upper-middle income)	1.5–2.5	Uneven, region-specific	Moderate	Urban concentration; economic instability affects staffing

## Conclusions

This review shows that pediatrics, once central to public health progress, is now at a point of transition. Despite major achievements in reducing infectious disease mortality and improving nutrition, the specialty faces declining student interest, workforce shortages, and significant disparities in compensation across all regions. These trends point to a growing mismatch between the need for pediatric expertise and the systems that support it. The evidence highlights a clear paradox. Success in controlling infectious diseases has shifted the pediatric burden toward chronic illness, developmental and behavioral disorders, and child mental health. These emerging challenges require skills that are not yet fully embedded in traditional training or practice models. Comparative findings from India, other low- and middle-income countries, and high-income settings show that the workforce crisis is global. Pediatric services remain undervalued, unevenly distributed, and inadequately supported. Compensation gaps and limited access to training in fields such as digital health, genomics, and population-based care continue to limit the specialty’s growth.

Pediatrics must now evolve from a reactive model of care to a proactive, integrative approach that reflects contemporary child health realities. Educational reform should focus on mental health, chronic disease prevention, and social determinants of health. Policy initiatives must establish fair reimbursement systems, expand training capacity, and invest in research that links pediatric care with measurable outcomes. Professional bodies have a responsibility to lead advocacy and build stronger partnerships across health systems. The future of pediatrics depends on collective commitment to renewal. If the specialty can realign its priorities, strengthen its workforce, and adapt its methods to the realities of modern child health, it can restore its influence and reaffirm its essential purpose: to safeguard the wellbeing and potential of every child.

## References

[1] Bansal CP, Gupta S (2013). The past half century of Indian Academy of Pediatrics (IAP). Indian Pediatr.

[2] Janssen DF (2023). Pediatrics: naming a medical specialty (1721-1880). Matern Child Health J.

[3] Dhaded SM, Somannavar MS, Moore JL (2020). Neonatal deaths in rural Karnataka, India 2014-2018: a prospective population-based observational study in a low-resource setting. Reprod Health.

[4] Bhutia DT (2014). Protein energy malnutrition in India: the plight of our under five children. J Family Med Prim Care.

[5] (2025). Levels and trends in child mortality. https://data.unicef.org/resources/levels-and-trends-in-child-mortality-2024/.

[6] Madan EM, Frongillo EA, Unisa S (2020). Effect of differences in month and location of measurement in estimating prevalence and trend of wasting and stunting in India in 2005-2006 and 2015-2016. Curr Dev Nutr.

[7] James KS, Singh SK, Lhungdim H, Shekhar C, Dwivedi LK, Pedgaonkar S, Arnold F (2021). National Family Health Survey (NFHS-5), 2019-21. https://dhsprogram.com/pubs/pdf/FR375/FR375.pdf.

[8] Lahariya C (2014). A brief history of vaccines & vaccination in India. Indian J Med Res.

[9] Awofeso N, Rammohan A, Iqbal K (2013). Age-appropriate vaccination against measles and DPT-3 in India - closing the gaps. BMC Public Health.

[10] Freed GL, Dunham KM, Jones MD Jr, McGuinness GA, Althouse L (2009). General pediatrics resident perspectives on training decisions and career choice. Pediatrics.

[11] Frintner MP, Cull WL (2012). Pediatric training and career intentions, 2003-2009. Pediatrics.

[12] Joe W, Kumar A, Rajpal S, Mishra US, Subramanian SV (2020). Equal risk, unequal burden? Gender differentials in COVID-19 mortality in India. J Glob Health Sci.

[13] Puri P, Khan J, Shil A, Ali M (2020). A cross-sectional study on selected child health outcomes in India: quantifying the spatial variations and identification of the parental risk factors. Sci Rep.

[14] Kalia M, Sharma M, Rohilla R, Rana K (2024). Trend of immunization & gap in vaccine doses as observed in National Family Health Survey rounds in India. Indian J Med Res.

[15] Biswas AB, Mitra NK, Nandy S, Sinha RN, Kumar S (2000). Missed opportunities for immunisation in children. Indian J Public Health.

[16] Mathew JL (2012). Inequity in childhood immunization in India: a systematic review. Indian Pediatr.

[17] Li W, Gillies RM, Liu C (2023). Specialty preferences of studying-abroad medical students from low- and middle-income countries. BMC Med Educ.

[18] Srinivasaraghavan R, Parameswaran N (2025). Pediatric practice in the present era - is a generalist still relevant in office and academic institutions?. Indian J Pediatr.

[19] Mukherjee SB, Agrawal D, Mishra D (2021). Indian Academy of Pediatrics position paper on nurturing care for early childhood development. Indian Pediatr.

[20] Summan A, Nandi A, Schueller E, Laxminarayan R (2025). Top 10 highest paying careers for doctors in India 2025. Vaccine.

[21] Freed GL (2025). Concerns regarding the pediatric workforce: what are we missing?. Pediatr Res.

[22] Barrett Fromme H, Jackson K, Cook JA (2025). Insights into the pediatric hospital medicine workforce: 2024 data and recent trends for programs. Hosp Pediatr.

[23] (2025). Results and data: 2024 main residency match. Data.

[24] Orr CJ, McCartha E, Vinci RJ (2024). Projecting the future pediatric subspecialty workforce: summary and recommendations. Pediatrics.

[25] Macy ML, Leslie LK, Turner A, Freed GL (2021). Growth and changes in the pediatric medical subspecialty workforce pipeline. Pediatr Res.

[26] Catenaccio E, Rochlin JM, Simon HK (2021). Differences in lifetime earning potential between pediatric and adult physicians. Pediatrics.

[27] Catenaccio E, Rochlin JM, Simon HK (2021). Differences in lifetime earning potential for pediatric subspecialists. Pediatrics.

[28] (2025). Physician compensation report 2025. https://www.doximity.com/reports/physician-compensation-report/2025.

[29] Courtwright SE, Barr EA (2023). Pediatric nurse practitioner workforce shortage threatens child health equity: key contributors and recommendations. J Am Assoc Nurse Pract.

[30] Dammann CE, Alvira CM, Devaskar SU (2025). Pediatric subspecialty workforce: what is needed to secure its vitality and survival?. Pediatr Res.

[31] Zhang X, Carabello M, Hill T, He K, Friese CR, Mahajan P (2019). Racial and ethnic disparities in emergency department care and health outcomes among children in the United States. Front Pediatr.

[32] Shipman SA, Wendling A, Jones KC, Kovar-Gough I, Orlowski JM, Phillips J (2019). The decline in rural medical students: a growing gap in geographic diversity threatens the rural physician workforce. Health Aff (Millwood).

[33] Rimsza ME, Ruch-Ross HS, Clemens CJ, Moskowitz WB, Mulvey HJ (2018). Workforce trends and analysis of selected pediatric subspecialties in the United States. Acad Pediatr.

[34] Jones S, Walter M (2023). Shortages of Care and Medical Devices Affecting the Pediatric Patient Population. https://www.ncbi.nlm.nih.gov/books/NBK596745/.

[35] (2025). Medical staffing in the NHS. https://www.bma.org.uk/advice-and-support/nhs-delivery-and-workforce/workforce/medical-staffing-in-the-nhs..

[36] (2025). Occupation shortage list. https://www.jobsandskills.gov.au/data/occupation-shortage/occupation-shortage-list.

[37] (2025). Australia’s health 2024: in brief. Australia’s Health.

[38] (2025). Comparing your pay against your peers: Medscape physician compensation report 2025. https://login.medscape.com/login/sso/getlogin?sc=ng&wcode=102&client=205502&scode=msp&urlCache=aHR0cHM6Ly93d3cubWVkc2NhcGUuY29tL3NsaWRlc2hvdy8yMDI1LWNvbXBlbnNhdGlvbi1vdmVydmlldy02MDE4MTAz.

[39] Lakshminrusimha S, Cheng TL, Leonard MB, Devaskar SU, Vinci RJ, Degnon L, St Geme JW 3rd (2024). Raising the bar: the need for increased financial support to sustain and expand the community of pediatric subspecialists. J Pediatr.

[40] (2025). 2024 Pediatrician salary report: lower pay leads many to supplement their income. https://weatherbyhealthcare.com/blog/pediatrician-salary-2024.

[41] Frintner MP, Sisk B, Byrne BJ, Freed GL, Starmer AJ, Olson LM (2019). Gender differences in earnings of early- and midcareer pediatricians. Pediatrics.

[42] Houtrow AJ, Zigler CK, Pruitt DW (2020). The state of the field: results from the 2014 and 2017 pediatric rehabilitation medicine practice surveys. PM R.

[43] Patel M, Raphael JL (2023). Pediatric subspecialty pipeline: aligning care needs with a changing pediatric health care delivery environment. Pediatr Res.

[44] Venkateswaran A, Singh AK (2025). India's health status and emerging priorities. Centre for Social and Economic Progress, New Delhi, India.

[45] Krishnaswami S, Nwomeh BC, Ameh EA (2016). The pediatric surgery workforce in low- and middle-income countries: problems and priorities. Semin Pediatr Surg.

[46] Ahmed MM, Oweidat M, Okesanya OJ, Alaswad M, Abdelbar SM, Gill P, Alsabri M (2025). Barriers to pediatric emergency care in low-resource settings: a narrative review. Sage Open Pediatr.

[47] Leeb RT, Danielson ML, Claussen AH (2024). Trends in mental, behavioral, and developmental disorders among children and adolescents in the. US, 2016-2021. Prev Chronic Dis.

[48] Xiang AH, Martinez MP, Chow T (2024). Depression and anxiety among US children and young adults. JAMA Netw Open.

[49] Racine N, McArthur BA, Cooke JE, Eirich R, Zhu J, Madigan S (2021). Global prevalence of depressive and anxiety symptoms in children and adolescents during COVID- 19: a meta-analysis. JAMA Pediatr.

[50] Ziebold C, McDaid D, King D (2025). Estimating the economic impacts for caregivers of young people with mental health problems in a Brazilian Cohort. Value Health.

[51] Bor W, Dean AJ, Najman J, Hayatbakhsh R (2014). Are child and adolescent mental health problems increasing in the 21st century? A systematic review. Aust N Z J Psychiatry.

[52] Sielaty R, Boutzoukas AE, Zimmerman KO (2023). Trends in pediatric emergency and inpatient healthcare use for mental and behavioral health among North Carolinians during the early COVID-19 pandemic. J Pediatric Infect Dis Soc.

[53] Hacker KA, Myagmarjav E, Harris V, Suglia SF, Weidner D, Link D (2006). Mental health screening in pediatric practice: factors related to positive screens and the contribution of parental/personal concern. Pediatrics.

[54] Di Cesare M, Sorić M, Bovet P (2019). The epidemiological burden of obesity in childhood: a worldwide epidemic requiring urgent action. BMC Med.

[55] Kumar S, Kelly AS (2017). Review of childhood obesity: from epidemiology, etiology, and comorbidities to clinical assessment and treatment. Mayo Clin Proc.

[56] Simmonds M, Burch J, Llewellyn A (2015). The use of measures of obesity in childhood for predicting obesity and the development of obesity-related diseases in adulthood: a systematic review and meta-analysis. Health Technol Assess.

[57] Sahoo K, Sahoo B, Choudhury AK, Sofi NY, Kumar R, Bhadoria AS (2015). Childhood obesity: causes and consequences. J Family Med Prim Care.

[58] Wang Y, Lim H (2012). The global childhood obesity epidemic and the association between socio-economic status and childhood obesity. Int Rev Psychiatry.

[59] Engin A (2024). The definition and prevalence of obesity and metabolic syndrome: correlative clinical evaluation based on phenotypes. Obesity and Lipotoxicity.

[60] Lang JE, Bunnell HT, Hossain MJ (2018). Being overweight or obese and the development of asthma. Pediatrics.

[61] Papoutsakis C, Priftis KN, Drakouli M (2013). Childhood overweight/obesity and asthma: is there a link? A systematic review of recent epidemiologic evidence. J Acad Nutr Diet.

[62] Garrido-Miguel M, Cavero-Redondo I, Álvarez-Bueno C (2019). Prevalence and trends of overweight and obesity in European children from 1999 to 2016: a systematic review and meta-analysis. JAMA Pediatr.

[63] Jullien S, Carai S, Weber MW (2024). Addressing the growing burden of obesity, diabetes and asthma in children and adolescents: the role of primary health care and the WHO Pocket Book in Europe for a healthy future. Glob Pediatr.

[64] Eckleberry-Hunt J, Lick D, Boura J, Hunt R, Balasubramaniam M, Mulhem E, Fisher C (2009). An exploratory study of resident burnout and wellness. Acad Med.

[65] Starmer AJ, Frintner MP, Freed GL (2016). Work-life balance, burnout, and satisfaction of early career pediatricians. Pediatrics.

[66] Shanafelt TD, Balch CM, Bechamps G (2010). Burnout and medical errors among American surgeons. Ann Surg.

[67] Tawfik DS, Profit J, Morgenthaler TI (2018). Physician burnout, well-being, and work unit safety grades in relationship to reported medical errors. Mayo Clin Proc.

[68] Leslie LK, Mehus CJ, Hawkins JD (2016). Primary health care: potential home for family-focused preventive interventions. Am J Prev Med.

[69] Kearns GL, Abdel-Rahman SM, Alander SW, Blowey DL, Leeder JS, Kauffman RE (2003). Developmental pharmacology—drug disposition, action, and therapy in infants and children. N Engl J Med.

[70] Marcin JP, Shaikh U, Steinhorn RH (2016). Addressing health disparities in rural communities using telehealth. Pediatr Res.

[71] McSwain SD, Bernard J, Burke BL Jr (2017). American Telemedicine Association operating procedures for pediatric telehealth. Telemed J E Health.

